# Mechanical Behaviors in Janus Transition-Metal Dichalcogenides: A Molecular Dynamics Simulation

**DOI:** 10.3390/nano12111910

**Published:** 2022-06-02

**Authors:** Fan Yang, Jing Shang, Liangzhi Kou, Chun Li, Zichen Deng

**Affiliations:** 1 School of Mechanics, Civil Engineering and Architecture, Northwestern Polytechnical University, Xi’an 710072, China; fanyang@mail.nwpu.edu.cn (F.Y.); lichun@nwpu.edu.cn (C.L.); 2 School of Materials Science & Engineering, Shaanxi University of Science & Technology, Xi’an 710021, China; 3 School of Mechanical, Medical and Process Engineering, Queensland University of Technology, Brisbane, QLD 4000, Australia; liangzhi.kou@qut.edu.au; 4 Research & Development Institute of Northwestern Polytechnical University in Shenzhen, Shenzhen 518057, China

**Keywords:** Janus transition-metal dichalcogenides, mechanical tensile behaviors, molecular dynamics

## Abstract

In this work, molecular dynamics simulations are performed to investigate the mechanical properties of Janus WSSe and MoSSe monolayers considering the effects of structural anisotropy, temperature, and tensile strain rates. The results demonstrate that Janus WSSe and MoSSe monolayers show strong mechanical anisotropy under tension along the armchair and zigzag directions, respectively. This anisotropy displays distinct temperature dependence. When the coupled effects of the temperature and anisotropy are considered for the tensions along the zigzag direction, there is a transition of ductile-to-brittle failure in the Janus WSSe monolayer at the critical temperature range of 80~90 K due to the competition between atomic thermal vibrations and structural bending/wrinkles. This phenomenon is further confirmed by both stress–strain curves and structural evolutions of the systems. Finally, a strain rate hardening mechanism is found when various strain rates are applied, and it demonstrates that the Janus monolayer could maintain stable mechanical properties under different loading conditions. Our investigations provide a helpful reference for subsequent theoretical and experimental studies on the mechanical properties of Janus monolayer structures and could shed some light on the design of promising nanoscale functional devices based on Janus transition-metal dichalcogenides.

## 1. Introduction

Transition metal dichalcogenides (TMDs) [1], such as MoS_2_, MoSe_2_, and WS_2_, have received considerable attention because of their unique electrical [2,3,4], magnetic [5], optical [6], and mechanical properties [7,8,9] with promising applications in nano-electronics [10,11]. 2H-TMDs monolayers are direct-bandgap semiconductors with a visible observation range of 400~700 nm (3~1.8 eV) [12], which is suitable for optoelectronics applications. In order to manipulate the properties, in-plane strains have been demonstrated to be effective where there is a direct-to-indirect transition of bandgap at a strain of ~1% and a semiconductor-to-metal transition at a strain of ~10% [9]. In comparison to the conventional TMDs, the electronic properties of the Janus TMDs MXY, where M is a transition metal (such as Mo and W) and X and Y are different types of chalcogens (i.e., M = Mo, W; X or Y = S, Se, Te; X ≠ Y), have novel features giving rise to a vertical dipole moment [13] due to the structural symmetry-breaking [14]. Motivated by the successful experimental achievement of a Janus MoSSe monolayer in the 2H phase [15], unique and intriguing physical properties of more two-dimensional (2D) TMDs Janus materials have been discovered and predicted in theoretical results [7,14,16,17], such as the Rashba effect [18,19], Skymions [20], large piezoelectricity [21], novel optical properties [22], and water splitting [23], where the internal electric field is helpful for separating an electron–hole pair. Mechanical properties of Janus TMDs play crucial roles in the successful applications of these outstanding properties in different applied conditions. This persuaded researchers to involve them in the understanding of mechanical behaviors. Previously, the tensile and compressive loading subjected to traditional TMDs has been studied theoretically in Zhao and Jiang’s work [24,25,26]. They investigated plenty of interesting and significant mechanical results regarding tensile and buckling 2D TMDs. In particular, Jiang et al. [26,27] presented a parameterization of the Stillinger–Weber (SW) potential to the interatomic interactions within single-layer TMDs. However, a comprehensive understanding of the mechanical behaviors of Janus TMDs has not been revealed yet.

In the present work, we have conducted a systematical study to investigate the mechanical behaviors of Janus WSSe and MoSSe monolayers under various uniaxial tensile loading conditions by molecular dynamics (MD) simulations. We found that the mechanical properties of the Janus WSSe monolayer display distinct anisotropy of armchair and zigzag directions and are dependent on the temperature and the tensile strain rate. These theoretical results provide a helpful reference for devices’ design based on Janus monolayer materials.

## 2. Method and Technique Details

**Figure 1:**
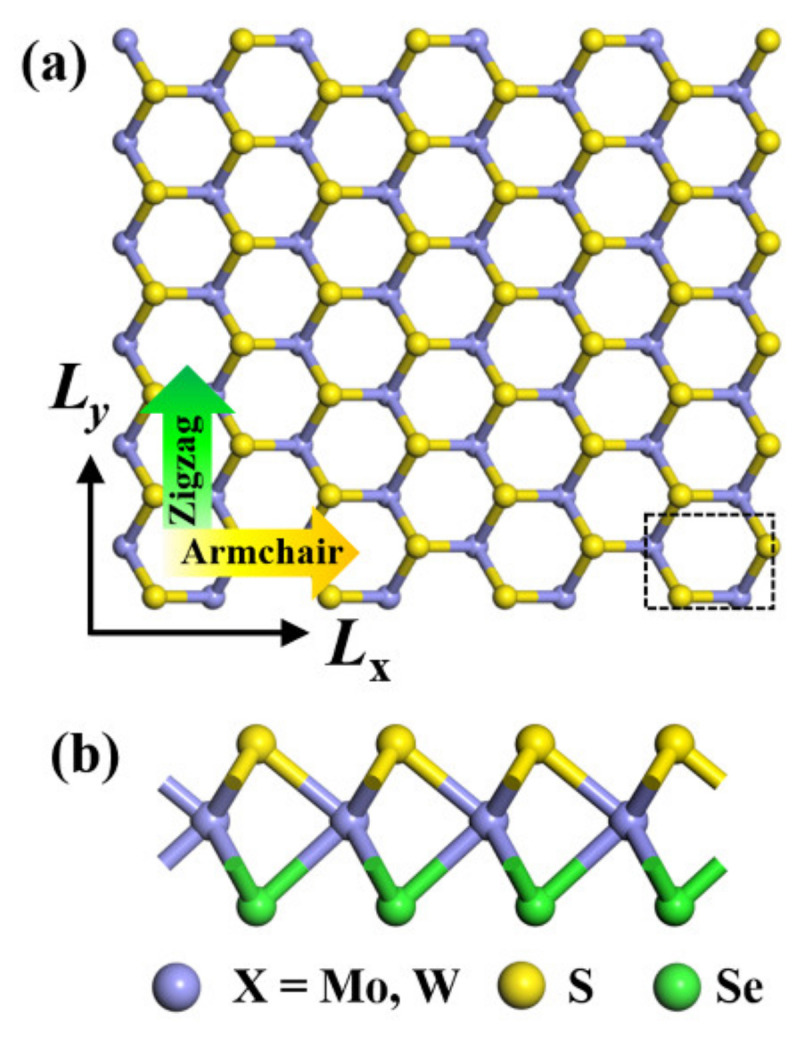
Top (**a**) and side (**b**) views of atomic structures of 2H–Janus transition-metal dichalcogenides monolayer with armchair (yellow arrow) and zigzag (green arrow) directions. The purple, yellow, and green balls represent X (X = Mo, W), S, and Se atoms, respectively.

In the present study, the 2D samples are constructed as 2H-phase Janus WSSe and MoSSe monolayer structures to perform MD simulations. The unit cell in a rectangular sample is shown in Figure 1a as a black dashed line. For Janus structures, top and bottom elements are S and Se, respectively, in the sandwich TMDs structure, as shown in Figure 1b. The initial structures of the Janus monolayers involve 50 × 50 supercells with 7500 atoms and sizes of 139.8 (*L_x_*) × 161.45 (*L_y_*) Å^2^ for both WSSe and MoSSe systems. To investigate the mechanical properties of Janus WSSe and MoSSe monolayers, external loading is applied along the armchair (*x*) and zigzag (*y*) directions, respectively (see Figure 1). The thicknesses for Janus WSSe and MoSSe are chosen as the effective thicknesses from their bulk structures, i.e., 8.10 and 8.04 Å for WSSe and MoSSe, respectively.

The MD simulations conducted in the present study are implemented using the open-source simulation code Large-scale Atomic/Molecular Massively Parallel Simulator (LAMMPS) [28] based on the standard Newton equations of motion. The periodic boundary conditions are applied along in-plane *x*, *y* orientations, and in the vertical *z*-direction; the thickness of the simulation box is set to be 50 Å to avoid the interaction between adjacent layers. The force interactions between atoms in the present Janus monolayers are described by the Stillinger–Weber (SW) potential with parameters provided by Jiang [26,27]. This potential has previously been proven to be reasonable for TMD systems [24,29,30]. The NPT (i.e., the number of atoms N, the pressure P, and the temperature T kept constant) ensemble is adopted for the whole simulation. In order to ensure the accuracy of the simulation, the velocity-Verlet algorithm is adopted, and the timestep is set as 0.001 ps for the energy minimization, the relaxation, and the tension process at specific temperatures. Before the structure is stretched, all the atoms are relaxed in the NPT ensemble for approximately 200 ps to erase the internal stress for x and y directions. To trigger the tensile simulations, the uniaxial tensile loading is applied with strain rates of 10^8^/s, 5 × 10^8^/s, and 10^9^/s, respectively, in the corresponding tensile conditions. The fracture processes of the Janus WSSe and MoSSe specimens are visualized by using the software OVITO [31]. During the tension simulations, the strain (*ε*) is defined as the change difference in the simulation box along the tension direction (armchair or zigzag), i.e., *ε* = (*l* – *l*_0_)/*l*_0_, where *l*_0_ and *l* are the initial and final lengths of the studied structure. The atomic stress tensor is calculated using the virial theorem implemented in LAMMPS, and the equations can be expressed as:

(1) $$\sigma_{t}\left(i\right)=-\overset{N}{\underset{j\neq \left(i\right)}{\sum }}f_{\alpha }\left(i,j\right)r_{\beta }\left(i,j\right)$$

(2) $$\sigma_{e}=-\overset{N}{\underset{i=1}{\sum }}\alpha_{t}\left(i\right)$$

where $\sigma_{t}\left(i\right)$ refers to the calculated stress for each atom. $f_{\alpha }$ is the interatomic force between atom i and j in direction α, $r_{\beta }$ is the distance in direction β. $\sigma_{e}$ in Equation (2) is the stress tensor for the overall structure, obtained by summing the atomic stress tensor obtained by Equation (1).

## 3. Results and Discussions

In this section, tensile tests based on MD simulations are performed to investigate the mechanical behaviors of Janus TMDs including MoSSe and WSSe monolayers under tensile conditions when considering their anisotropy of armchair and zigzag directions, temperatures, and tensile strain rate effects.

### 3.1. Anisotropy

**Figure 2:**
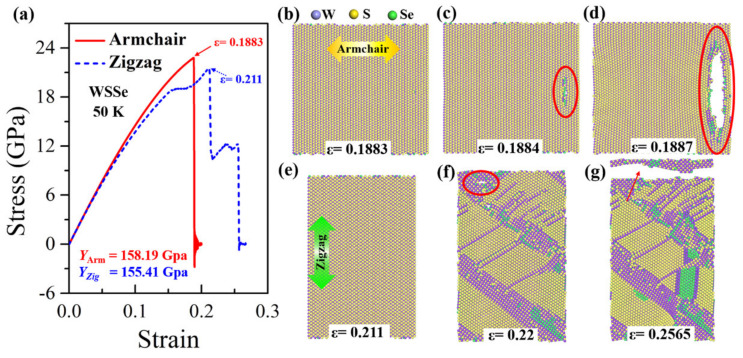
Anisotropy of tensile behaviors for Janus WSSe monolayer. (**a**) Stress–strain curves of Janus WSSe monolayer along armchair and zigzag loading directions at temperature of 50 K. (**b**–**g**) Fracture process of Janus WSSe monolayer along armchair (**b**–**d**) and zigzag (**e**–**g**) loading directions.

**Figure 3:**
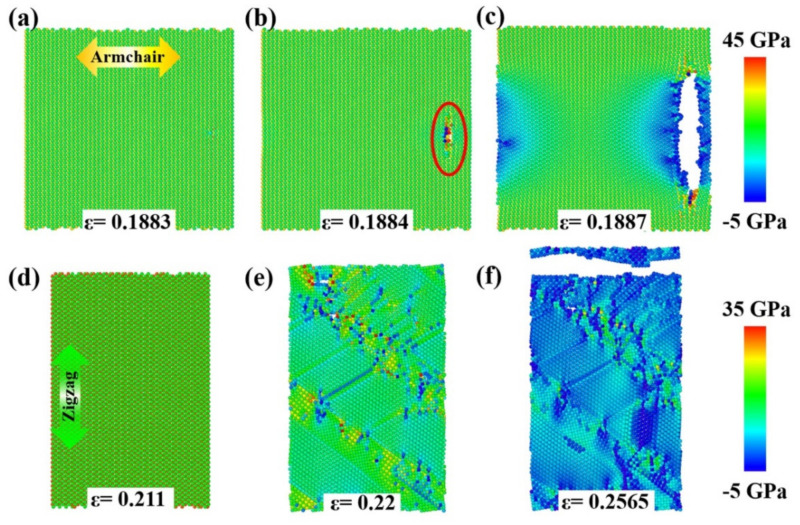
The stress distribution of the Janus WSSe monolayer sheet structure evolutions corresponding to the fracture process shown in Figure 2b–g. Tensile along the armchair (**a**–**c**) and zigzag (**d**–**f**) directions, both with a strain rate of 10^9^/s at 50 K.

In our calculations, the tensile anisotropy test is conducted in Janus MoSSe and WSSe monolayers by uniaxial stretching along the armchair and zigzag directions, respectively. The results show that the Janus WSSe monolayer displays the most distinct mechanical anisotropy at lower temperatures. The uniaxial stress–strain curves of the Janus WSSe monolayer at a temperature of 50 K are plotted in Figure 2a. Notably, the stress here in the stress–strain curve is the uniaxial stress along the tensile direction, obtained by summing the single atomic stress along the same direction. By fitting the stress–strain curves within the strain range of 0~0.03, the slopes of the elastic period (Young’s modulus *E* = Δ*σ*/Δ*ε*, where Δ*ε* is the strain increment and Δ*σ* is the corresponding stress increment) are denoted in the figure. It can be seen that the armchair structure shows a higher Young’s modulus of 158.19 GPa, compared to the zigzag case (155.41 GPa), which behaves differently from silicon-germanium nanotubes [32]. These values are smaller than that in a previous study [8]; the reason lies in that, in the present study, the adopted thickness values are larger than those in Zhong’s study, leading to a larger volume and thus smaller Young’s module. Moreover, the ultimate strength (the largest stress during the entire tensile process) along the armchair direction is 22.81 GPa, which is obviously higher than the corresponding value for the zigzag direction (21.49 GPa). Although the armchair structures exhibit a higher Young’s modulus and failure stress, in contrast, its ultimate strain extracted from stress–strain curves (*ε* = 0.1883) is much smaller than that of the zigzag structure (*ε* = 0.211), as shown in Figure 2a. More importantly the stress–strain curves show that the Janus WSSe monolayers exhibit different fracture modes, where they experience a brittle fracture with a sudden drop after the ultimate strain (*ε* = 0.1883) when the tension is applied along the armchair direction, and a ductile fracture with a constant stage after the first drop when the tensile loads are applied along the zigzag direction. This phenomenon is confirmed by the structural failure process as seen in Figure 2b–h, and the corresponding stress distributions are shown in Figure 3a–f.

**Figure 4:**
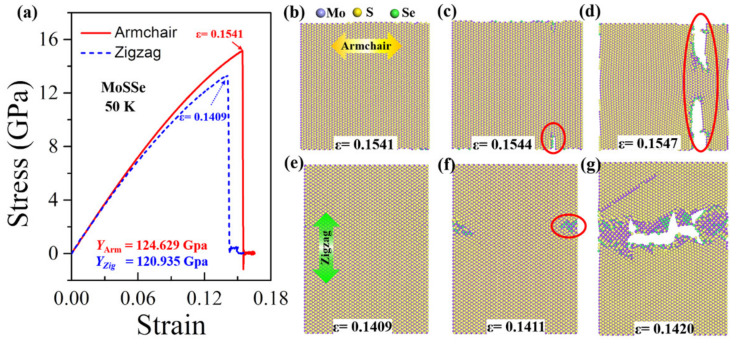
Anisotropy of tensile behaviors for Janus MoSSe monolayer. (**a**) Stress –strain curves of Janus MoSSe monolayer along armchair and zigzag loading directions at temperature of 50 K. (**b**–**g**) Fracture process of MoSSe monolayer along armchair (**b**–**d**) and zigzag (**e**–**g**) loading directions.

**Figure 5:**
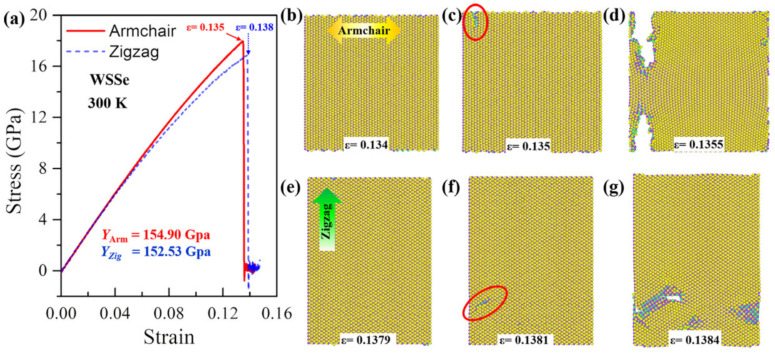
Anisotropy of tensile behaviors for Janus WSSe monolayer. (**a**) Stress–strain curves of Janus WSSe monolayer along armchair and zigzag loading directions at temperature of 300 K. (**b**–**g**) Fracture process of WSSe monolayer along armchair (**b**–**d**) and zigzag (**e**–**g**) loading directions.

In the armchair tensile condition, the elastic period lasts until the strain reaches 0.1883 (the ultimate strain), and no crack or void is originated. The corresponding stress distributes consistently in the monolayer structure, as shown in Figure 2b and Figure 3a, respectively. Under the following tensile load (*ε* = 0.1884), a crack appears at the right part of the WSSe nanosheet with the brittle breaking of S-W and Se-W bonds (see the red circles in Figure 2c and Figure 3b). It can be seen that the stress concentration and release occur around the broken S-W and Se-W bonds, represented by the high (red) and low (blue) stress distributions, respectively. Afterward, the crack grows fast and finally leads to a brittle fracture, as shown in Figure 2d and Figure 3c, and the stress declines quickly around the crack. In contrast, in the zigzag tensile case, after the instant of the ultimate strain of 0.211, some S-W or Se-W bonds break. However, in the further tensile process, W atoms still connect to maintain the whole structure as seen in Figure 2f within a strain range of 0.211 to 0.22 to experience a ductile stage. The stress decreases along the broken S-W or Se-W bonds as shown in Figure 3c. Then the crack appears due to the broken W-W bonds as labeled by the red circle in Figure 2f. In the subsequent tensile process, the structure undergoes a typical brittle fracture originating from the void as indicated by the red arrow in Figure 2g. It is worth mentioning that, in Figure 2c, the crack initiates from the interior of the monolayer rather than the edge because of the periodic boundary conditions adopted in the present simulation. Similar anisotropy phenomena of Young’s modulus have also been found in the stretched Janus WSSe structure under the temperature of 300 K and the Janus MoSSe monolayer under the temperature of 50 K, as seen in Figure 4 and Figure 5, respectively, but they all exhibit a brittle fracture. It should be noted that this anisotropy effect of fracture modes in WSSe monolayer systems with tension along the zigzag direction displays significant differences at higher temperatures (above 90 K) than that at lower temperature range. Therefore, in the following section, the temperature effect is considered and discussed in detail.

### 3.2. Temperature-Dependent Failure Modes

Temperature plays an important role in the mechanical properties of nanomaterials, especially for 2D layered materials. To investigate the thermal effect on the mechanical properties of Janus TMDs monolayers, taking the Janus WSSe monolayer as a typical example, ten different temperatures from 30 to 400 K have been adopted for Janus WSSe monolayer samples composed of 7500 atoms. As shown in Figure 5a, the Janus WSSe monolayer displays apparently distinct mechanical properties under different temperatures. As seen from the obtained values of ultimate strength (black) and the elastic modulus (blue), the mechanical strength of the Janus WSSe monolayer decreases with increasing temperature from 30 to 400 K, and the temperature range of 80~90 K exhibits some distinct phenomena. On the one hand, for the elastic modulus, although there exist some fluctuates before the temperature reaches 90 K, its decrease rate with respect to the temperature is relatively smooth, which is also consistent with the linear stage of the stress–strain curves shown in Figure 5b. On the other hand, there exists a sharp drop in the ultimate strength when the temperature is increased from 80 to 90 K, indicating that a possible transition of failure modes of the structure occurs within this temperature range. Similar temperature-dependent mechanical properties have also been confirmed in the single-layer graphene with grain boundaries [33]. This is closely related to the tensile fracture modes under different temperature conditions. More importantly, it is found that there is a transition from ductile (yellow zone) to brittle (pink zone) fracture modes between the temperature of 80 and 90 K presented in the stress–strain curves shown in Figure 5b. To clarify the stress–strain curves for the lower and higher temperature ranges, solid and dashed lines are presented for lower and higher temperature cases, respectively. Specifically, the Janus WSSe monolayer behaves as a ductile fracture at lower temperatures (30~80 K), but a brittle fracture mode when temperatures are higher than 90 K. This phenomenon could be confirmed by the corresponding fracture process of the Janus WSSe monolayer samples when applying different temperatures. Therefore, we take samples with these two critical temperatures (80 and 90 K) to discuss the corresponding fracture modes.

**Figure 6:**
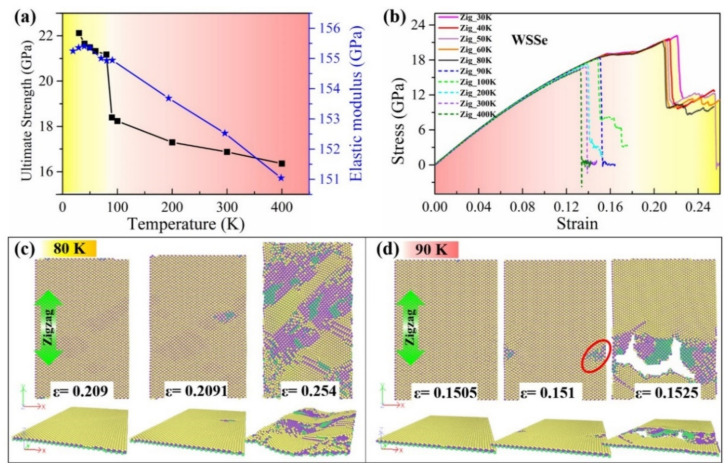
Temperature effect on mechanical properties of Janus WSSe monolayer with stretching along zigzag direction (denoted as “Zig”). (**a**) Ultimate stresses (black line) and elastic modulus (blue line) under different temperature conditions; (**b**) stress–strain curves under various temperatures from 30 to 400 K; (**c**,**d**) top view and side view of fracture process in WSSe monolayer under tensile loading along the zigzag direction with temperature 80 K (**c**) and 90 K (**d**). The purple, yellow, and green balls represent W, S, and Se atoms, respectively.

**Figure 7:**
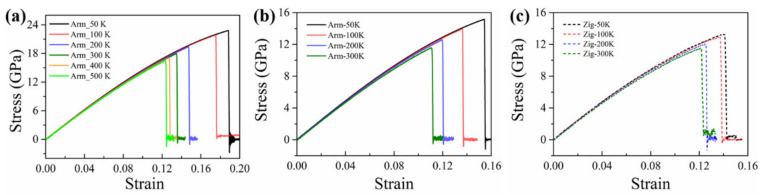
Stress–strain curves of Janus WSSe monolayer along armchair loading direction (**a**) and Janus MoSSe monolayer along armchair and zigzag loading directions (**b**–**c**) at the temperature range of 50 to 300 K.

Figure 6c displays the top and perspective views for the fracture process of the Janus WSSe monolayer with three typical strains (*ε* = 0.209, 0.2091, and 0.254) with a temperature of 80 K. At the elastic stage within the strain *ε* < 0.209, no fracture or crack initiation occurs. In the strain range of *ε* = 0.209 to 0.254, the Janus WSSe monolayer experiences a ductile deformation as shown in Figure 6c. During this stage, W-S and W-Se bonds break, but the skeleton structure composed of W atoms (purple atoms) is preserved so that it can undergo a ductile deformation until the whole system is damaged. It should be noted that when the stretching strain is applied along the zigzag direction, a wrinkled structure composed of W atoms can be observed along the armchair orientation as seen in the perspective view in Figure 6c to avoid a brittle fracture. In contrast, for the case of a temperature of 90 K, after the ultimate strain of 0.1505, the material goes straight into the brittle fracture when stretching along the zigzag direction (see Figure 6d). At the next instant of strain 0.151, a crack originates from the broken bonds of W-S and W-Se, as indicated in the red circle in Figure 6d. Afterward, with the tensile strain increasing to 0.1525, the 2D nanosheet breaks from the crack, leading to the final failure, corresponding to the stress drop in the stress –strain curve (blue dashed curve) in Figure 6d. In detail, during the tensile process, when the temperature is above 0 K, competition exists between the atomic thermal vibrations and the structural bending/wrinkles, which can induce breaks between atomic bonds and ductile behaviors, respectively. When the temperature is lower than 80 K, the atomic thermal vibrations are relatively weak regarding breaking the atomic bonds, and simultaneously, stretching along the zigzag direction results in the shrinking of the armchair direction to form wrinkles or bending deformations, as shown by *ε* = 0.254 in Figure 6c. Thus, after the next instant of the ultimate stress, the wrinkle structures contribute to tolerating the external stretch, so that it behaves in a ductile failure mode. On the contrary, when the temperature is above 90 K, the stronger atomic thermal vibration plays a more significant role in the fracture process, thus the atomic bonds prefer to break directly to form cracks under the tensile condition, as shown by *ε* = 0.1525 in Figure 5d. Therefore, when the temperature is increased, the fracture mode is changed from ductile to brittle. This transition from ductile to brittle failure of TMDs was also found in S-doped 2H-MoSTe ternary alloys, but with a different mechanism [8]. In addition, for the WSSe monolayer with tension along the armchair direction and the MoSSe monolayer under tensile strains along both armchair and zigzag directions, a brittle fracture without wrinkles is found within all temperature ranges, as seen in the stress–strain curves in Figure 7. This temperature-independent mechanical behavior of the Janus WSSe monolayer will not only expand the new properties of 2D mechanics in Janus materials but also provide guidelines for future experiments and device applications.

### 3.3. Strain Rate Effect

For the tensile loading, the strain rate plays an important role in affecting the strength of nanomaterials, including a one-dimensional (1D) nanotube [32,34], 2D sheets [35], three-dimensional (3D) metals [36,37], and high-entropy alloys [38]. To gain insight into the coupled effect of the tensile strain rate, loading direction, and temperature on the fracture strength, additional simulations have been performed on Janus WSSe and MoSSe monolayers at different temperatures ranging from 50 to 300 K with different strain rates of 10^8^/s, 5 × 10^8^/s, and 10^9^/s.

**Figure 8:**
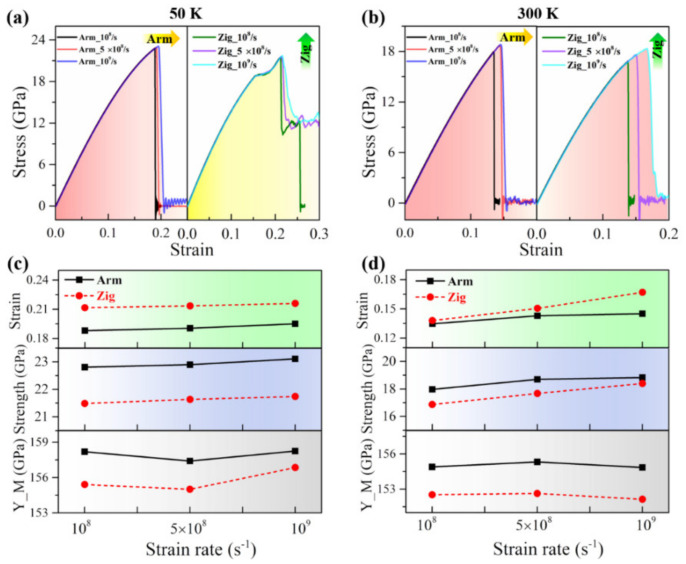
Tensile strain rate effect on mechanical properties of the Janus WSSe monolayer. (**a**–**b**) Stress–strain curves when extended along armchair (left panel, denoted as “Arm”) and zigzag (right panel, denoted as “Zig”) directions under various tensile strain rates at 50 K (**a**) and 300 K (**b**). (**c**–**d**) The ultimate strain (upper panel: Strain), ultimate strength (middle panel: Strength), and Young’s modulus (lower panel: Y_M) with various tensile strain rates at 50 K (**c**) and 300 K (**d**).

Taking the temperatures of 50 and 300 K as typical examples, the simulation results of the stress–strain relation, the ultimate strain, the ultimate strength, and Young’s modulus of the Janus WSSe monolayer are depicted in Figure 8. At lower temperatures (50 K), the fracture modes remain the same at various tensile strain rates, where armchair and zigzag tensions are brittle (left panel: Red area) and ductile (right panel: Yellow area) failure conditions, respectively, as seen from Figure 8a. However, certain parameters such as the ultimate strain, the ultimate strength, and Young’s modulus during tension change obviously, as shown in Figure 8c. With the tensile strain rate increasing, the ultimate strain and strength increase for both armchair and zigzag cases due to the strain rate hardening mechanism. Compared to the zigzag case, the armchair structures exhibit smaller ultimate strains but larger ultimate strengths. However, Young’s modulus declines under a strain rate from 10^8^/s to 5 × 10^8^/s and then rises when the strain rate increases to 10^9^/s. The same trends are also found in higher temperature conditions, as shown in Figure 8b,d. Note that Young’s modulus behaves differently for the Janus WSSe monolayer, as it increases under a strain rate of 10^8^/s to 5 × 10^8^/s and then decreases when the strain rate increases to 10^9^/s. Overall, the mechanical behaviors of the Janus WSSe monolayer are related to the tensile strain rate, but the mechanical parameters do not change significantly under various strain rates, which provides evidence that this 2D Janus monolayer possesses steady mechanical properties under various tensile load conditions.

**Figure 9:**
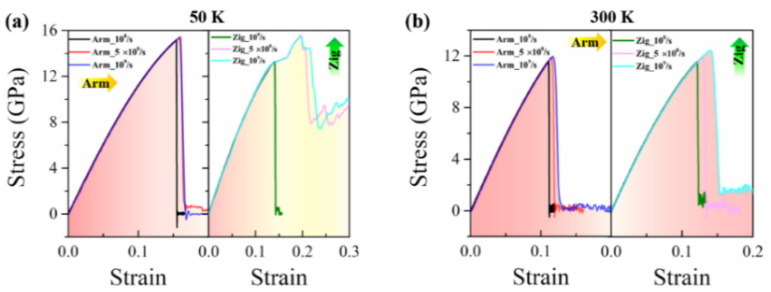

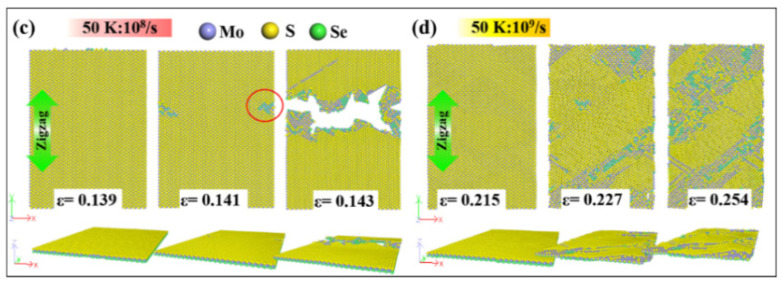
Tensile strain rate effect on mechanical properties in Janus MoSSe monolayer. (**a**–**b**) Stress–strain curves when extended along armchair (left panel, denoted as “Arm”) and zigzag (right panel, denoted as “Zig”) directions under various tensile strain rates at 50 K (**a**) and 300 K (**b**). (**c**–**d**) Top view and side view of fracture process in MoSSe monolayer under tensile loading along the zigzag direction with temperature of 50 K and strain rate of 10^8^/s (**c**) and 50 K and strain rate of 10^8^/s (**d**). The purple, yellow, and green balls represent Mo, S, and Se atoms, respectively.

Regarding the Janus MoSSe monolayer, at the lower temperature of 50 K, it behaves in distinct strain-rate-dependent fracture modes when the tensile load is applied along the zigzag direction, as shown in the right panel of Figure 9a. At a relatively low strain rate, the failure prefers a brittle fracture with a sudden drop in stress, as the green curve shows. Accordingly, the tensile process shows that once a crack forms at strain *ε* = 0.141, the whole structure breaks immediately, as seen in Figure 9c. Meanwhile, in higher strain rate conditions (5 × 10^8^/s and 10^9^/s), there is a ductile period after the ultimate strains, and the corresponding structural evolutions are shown in Figure 9d. In addition, a wrinkle can also be found when the strain reaches *ε* = 0.254. For the armchair case at 50 K and both cases at 300 K, this material tends to show a brittle failure, as shown in the left panel of Figure 9a–b.

## 4. Conclusions

In summary, the mechanical behaviors of Janus WSSe and MoSSe monolayers are systematically investigated considering the anisotropy of armchair and zigzag directions, temperatures, and tensile strain rate effects using MD simulations. The obtained results show that, firstly, when the tensile strains are applied along the armchair and zigzag directions, respectively, WSSe and MoSSe monolayers show strong mechanical anisotropy, and this anisotropy is temperature-dependent. Secondly, when considering the coupling effect of temperature and structural anisotropy, the WSSe monolayer exhibits different failure modes with temperature under the condition of stretching along the zigzag direction, that is, it experiences ductile failure at temperatures below 90 K, while it changes to a brittle failure mode at temperatures above 90 K. This phenomenon results from the competition between atomic thermal vibrations and structural bending/wrinkles and is further confirmed by the stress–strain curve and structural evolution of the system. Finally, the strain rate hardening mechanism under different strain rates is found, which shows that Janus monolayer structures can maintain stable mechanical properties under different loading conditions. The present simulation results are expected to provide a useful reference for subsequent theoretical and experimental research on the mechanical properties of Janus monolayer structures and provide a theoretical basis and new ideas for the design of nano functional devices based on Janus TMDs.
